# Wireless-controlled cubic neural stimulator for free-moving animals

**DOI:** 10.1098/rsos.221116

**Published:** 2023-03-01

**Authors:** Xinyu Liu, Zhenling Su, Qingran Gao, Yanna Ping, Hang Xie, Yang Yang, Dongyun Wang

**Affiliations:** ^1^ School of Intelligent Manufacturing, Huanghuai University, Zhumadian 463000, People's Republic of China; ^2^ School of Control Science and Engineering, Dalian University of Technology, Dalian 116024, People's Republic of China; ^3^ School of Mechanical and Electrical Engineering, Jiyuan Vocational and Technical College, Jiyuan 459000, People's Republic of China; ^4^ Department of Neurosurgery, Zhumadian Central Hospital, Zhumadian 463000, Henan, People's Republic of China

**Keywords:** neural stimulator, flexible printed circuit board, free-moving animal, electrical stimulation

## Abstract

An electrical stimulator transmitting information into selected neural circuits is a promising approach for neural prostheses or animal robots. However, traditional stimulators are based on rigid printed circuit board (PCB) technology; technological limitations hindered the development of stimulators, especially for experiments involving free-moving subjects. Here we described a small (1.6 × 1.8 × 1.6 cm), lightweight (4 g, including a 100 mA h lithium battery) and multi-channel (eight unipolar or four bipolar biphasic channels) cubic wireless electrical stimulator exploiting flexible PCB technology. In comparison with the traditional stimulator, an appliance of both flexible PCB and cube structure makes it smaller and lighter, and enhances its stability. Stimulation sequences can be constructed with 100 selectable current levels, 40 selectable frequency levels and 20 selectable pulse-width-ratio levels. Moreover, the distance of wireless communication can reach approximately 150 m. Both *in vitro* and *in vivo* results have demonstrated functionality of the stimulator. The feasibility of remote pigeon's navigation using the proposed stimulator was successfully verified.

## 1. Introduction

Electrical stimulation of neural circuits, which initiates a functional response by depolarizing the membranes of excitable cells that can elicit and modify neuronal activities, has a long application history. It has been applied to neuroscience research and clinical therapies as well as extending to targets in the peripheral and central nervous system [1,2]. For example, both cochlear implant and visual prosthesis are well established for direct stimulation of sensory afferents [3,4], and electrical stimulation to targets on the central nervous system has also been achieved for the treatment of chronic pain and central motor disorders, such as tremor in Parkinson's disease, with deep brain stimulation [5]. Thus, it has a wide application prospect for the treatment of neurological diseases [6]. In addition, that sensory perception could be also biased by external electrical stimulation and significantly broaden horizons in various research areas [7–9].

Electrical stimulation is generally realized depending on an external stimulator [10], which produces biphasic current pulses to achieve an overall zero net charge. However, traditional stimulators always require a set of electrical wires to realize their connection with experimental subjects. Despite being free from the restriction of size and weight as well as more accurate pulses output, its scope-of-use multiple is greatly compressed by practical limitations, especially for free-moving animals. For example, the application of wires not only limits the motion range of experiment subjects, but also are easy to be tangled or twisted [11], which can even distract the subject's attention or produce emotional distress [12], increasing the complexity of experimental design and interfering with experimental results. Although the dedicated device, such as a commutator [13], can offset this effect to a certain extent, its role is extremely limited for free-moving animals.

Establishing a wireless system to communicate with stimulators is a common solution [14–16]; however, wireless stimulators have quite strict requirements for size and weight, where the weight of the stimulator cannot exceed 5% of self-weight in order to avoid affecting the normal activity of animals [17], especially for flying birds, and an irreconcilable contradiction exists between power supply and communication distance as well as the size and weight for the wireless stimulator. Although some authors have successfully remotely delivered electrical pulses to the brain of free-moving animals [14–16,18,19], the size and weight limit the application and practical usage of these devices in experiments. Therefore, there lacks a wireless stimulator with appropriate weight and size adapted for chronic stimulation in small free-moving animals.

The application of specific integrated circuits and implantable neural stimulators are two main approaches for wireless stimulators, which have the advantages of reduced size and lower power consumption capabilities. However, the high economic and time cost as well as the narrow application range of the integrated circuits deters many studies, which tend to be reserved for high-volume products. For implantable neural stimulators [6,9], small and distributed neural dust have eliminated the requirement for leads, batteries and a centralized stimulation unit, but superb surgical techniques and extremely short wireless distance cannot fit the free-moving animals. Alternatively, fully implantable systems with a centralized stimulator and embedded battery [20–22], which albeit improve communication distance, must be placed in a spacious anatomical location, and the high failure rate often burdens the practical applications [10].

Here we propose a small (1.6 × 1.8 × 1.6 cm), light in weight (4 g) and multi-channel (eight-channel) wireless electrical stimulator for free-moving animals, with flexible printed circuit board (PCB) technology applied to print the circuit board and a cube structure designed with the battery inside and the circuit outside, which greatly reduce the size and weight of stimulator. Here the ZigBee module was used for wireless communication. Large communication distance, small volume and light weight make it suitable for most laboratory free-moving animals. More importantly, the stimulator made of ordinary electronic components has a low cost, making it acceptable to most laboratories. The detail of the proposed stimulator is described in the following sections.

## 2. Material and methods

The purpose of this study was to design and construct a neural stimulator that can perform electrical stimulation for free-moving animals, which is composed of two parts: a remote-controlled stimulator to generate stimulation pulses, and a handheld controller to send stimulation commands to the stimulator. Although the controller is also crucial for the whole system, it is not the focus of this study and will not be detailed here.

### 2.1. Structure and functional description

**Figure 1.**
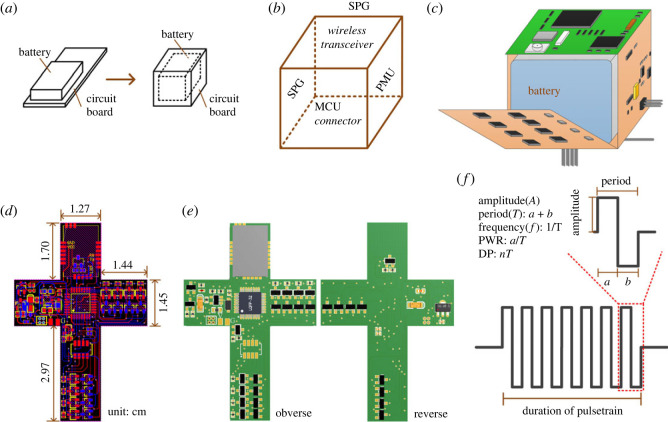
Overview of the proposed neural stimulator. (*a*) Comparison between the traditional stimulator and the proposed stimulator. (*b*) Function distribution of the proposed stimulator. SPG: stimulation pulse generator; MCU: microcontroller unit; PMU: power management unit. (*c*) Schematic of the proposed stimulator. (*d*) Layout and size of the proposed stimulator. (*e*) Distribution of electronic components on the obverse and reverse of stimulator. (*f*) Schematic of the output waveforms. *T*: period, *T* = *a* + *b*; *a*: positive phase width; *b*: negative phase width; *f*: frequency, *f* = 1/*T*; PWR: phase-width ratio, PWR = *a*/*T*; DP: duration of pulse train, DP = *nT*, *n* is a integer (*n* > 0).

A conceptual overview of the proposed stimulator is depicted in figure 1. It is generally composed of a circuit board and battery, where the battery is inside and the circuit board is outside for our stimulator to form a cube structure, compared with traditional stimulators, in which the circuit board and the battery are stacked (figure 1*a*). The cube structure can make the electronic components more compact and thus reduce the volume of the device. In addition, the circuit board is made depending on flexible PCB technology so that it can be bent arbitrarily. In the circuit board, different modules are distributed on different sides of the cube according to their functions (figure 1*b*), where the top is the wireless transceiver, the bottom is the eight-channel connector, the right and the back are the two four-channel stimulation pulse generators (SPG), the left is the power management unit, and the front is the microcontroller unit (MCU). Figure 1*c* shows the rendered graph of the stimulator. To realize the above cube structure, a cross-shaped flexible circuit board was designed (figure 1*d*). The front and back sides of the rendered circuit board are shown in figure 1*e*. The stimulator is assembled by a process similar to folding a box. The size of the circuit board is shown in figure 1*d*.

The function of the stimulator is to output electrical stimulation pulse waveform (figure 1*f*). In order to reduce the damage to nerve tissue caused by stimulation, the stimulation waveform generally consists of positive pulse and negative pulse. Amplitude, frequency, phase width and duration of pulse train are the four core parameters of the stimulation pulse. If the positive phase width is *a* and the negative phase width is *b* in a period (*T*), then *T* = *a* + *b*. The stimulation frequency (*f*) can be expressed as *f* = 1/*T*. Because the width of the phase width is determined by the stimulation frequency (*f*), we do not use the traditional phase-width index here, but introduce a new index, phase-width ratio (PWR), which is defined as the ratio of the width of the positive phase width (*a*) to the width of the pulse period (*T*), i.e. PWR = (*a*/*T*) × 100%. The duration of pulse train (DP) is *nT*, *n* is a integer (*n* > 0). The greater the *n*, the longer the DP of the pulse. The DP is determined by the battery capacity. As long as the battery capacity of the stimulator is sufficient, the pulse can be output all the time. So the DP is not generally discussed separately.

### 2.2. System and circuit design

**Figure 2.**
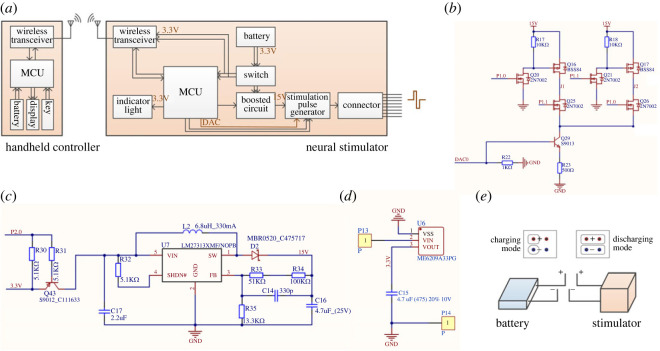
Circuit schematic of the proposed neural stimulator. (*a*) Block diagram of the stimulation system. MCU: microcontroller unit. (*b*) An independent current-generating circuit, two channels and one current source are shown (from eight channels and two independent current sources). (*c*) Boosted circuit. (*d*) Power supply circuit. (*e*) Switch between charging and discharging modes.

A block diagram of the neural stimulation system illustrates the composition of the handheld controller and neural stimulator (figure 2*a*). For the handheld controller, stimulation parameters are input through a keyboard and written to a liquid crystal display after being processed by the MCU. After the setting is completed, the parameters are sent to the stimulator through a wireless transceiver. For the neural stimulator, after the transceiver received parameters, both the SPG and the boost circuit are controlled by the MCU to generate the corresponding stimulation pulse, which is a biphasic electrical current pulse. The function of the boost circuit is to maintain the output current constant to avoid it changing due to different load. The stimulator is capable of delivering biphasic current pulses through the connector to four addressable electrode sites, in which the indicator light will be lit when the stimulator is powered on or outputs a stimulation pulse.

C8051F410 chip was used as the main control chip for the MCU in both the handheld controller and neural stimulator, which was equipped with two 12-bit current mode digital-to-analogue converters, of which the output current signal is converted into the required stimulation voltage through the constant current/constant voltage conversion circuit to provide stimulation pulse for each channel. A boost circuit, in which LM 2371 chip was adopted, was designed to increase the system voltage from 3.3 to 15 V to supply power for the conversion circuit (figure 2*c*). Each stimulation site is composed of two channels, which switch to generate biphasic pulse (figure 2*b*). A commercial ZigBee module (Z151, Ghostyu Co., Ltd, China) was applied to realize wireless communication between the handheld controller and neural stimulator, which transmits the signal by a 2.4 GHz radio frequency transceiver.

The working voltage of the stimulator is 3.3 V powered by a lithium battery, the circuit is shown in figure 2*d*. Due to the size constraints, two 3.7 V, 50 mA h batteries are connected in parallel serving as power supply. To conserve power and reduce the size, a self-made switch is designed in addition to selecting low-power devices (figure 2*e*), where a four-channel connector is used, with two channels connected to the positive and negative poles of the battery, and the other two channels connected to that of stimulator. When the stimulator is supposed to be powered on, two jump caps are applied to connect their positive and negative poles, respectively. In circuit board printing, flexible PCB technology was adopted to print the circuit board.

### 2.3. Fabrication and encapsulation

**Figure 3.**
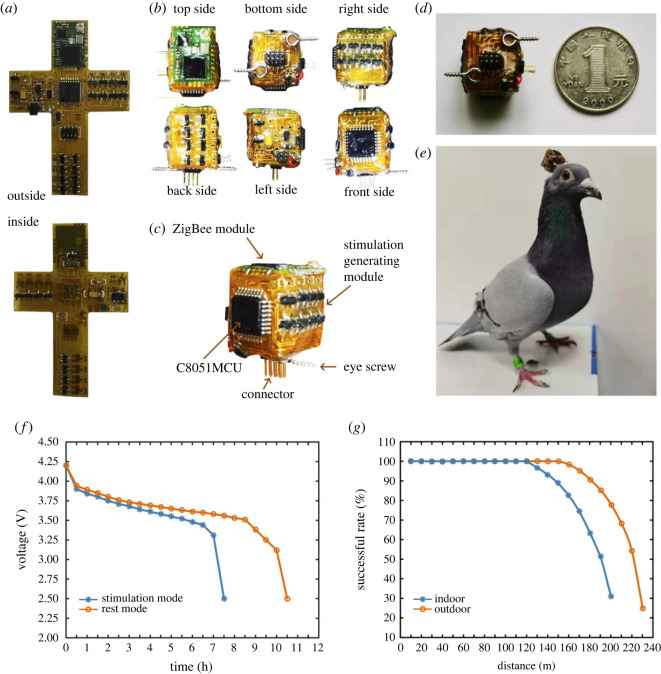
Fabrication of the proposed neural stimulator. (*a*) Photograph of the circuit board outside and inside. (*b*) Different view of stimulator. (*c*) Photograph of stimulator. (*d*) Size comparison between the stimulator and a RMB coin (1 yuan). (*e*) Photograph of the pigeon with a stimulator. (*f*) Voltage consumption during different modes. (*g*) Successful rate of communication at different distances.

All electronic components were soldered with tin wire, with three-proofing lacquer coating on the circuit board, which should be cleaned with detergent followed by isopropyl alcohol prior to encapsulation [22]. As the coating of three-proofing lacquer is very thin, the bending of the circuit board will not be affected. The circuit board with protective coating is shown in figure 3*a*. After the coating was cured, the circuit board was folded to be a folding box, with the battery (lithium battery, 100 mA h) placed inside. Due to the flexible PCB being easy to damage, it must be encapsulated with glue before use. So the stimulator was encapsulated and fixed with epoxy resin AB glue (Kafuter, Hengdr Co., Ltd, China), which is allowed to cure at room temperature (about 2 h). Different components were distributed on the six sides of the stimulator referring to their functions (figure 3*b*), where a ZigBee communication module was placed above, and both the eight-channel connector and two horn screws were located below, for fixing the stimulator with the electrode on the animal head (figure 3*c*). The stimulator had an eight-channel interface that can realize independent stimulation at four sites.

### 2.4. *In vitro* performance testing

In the *in vitro* performance testing, a resistance (10 kΩ) is first attached at the output end of the stimulator, the commands with different parameters are sent through the handheld controller, and biphasic pulses generated from the stimulator are measured by an oscilloscope (GDS-1102B, Good Will Instrument Co., Ltd, China) to examine the accuracy of the output pulse of the stimulator. Secondly, an electrode was dipped in phosphate-buffered saline (PBS) solution (biosharp BL302A, Labgic Technology Co., Ltd, China) to mimic the *in vivo*-like environment [23], with connections to the stimulator. As in the above steps, its performance is assessed by measuring its output pulses.

### 2.5. *In vivo* performance testing

In the *in vivo* performance testing, two classical experiments were conducted, i.e. stimulus-evoked potentials detection experiment (namely the detection experiment) and stimulus-induced behaviour regulation experiment (namely the regulation experiment), where three adult pigeons (*Columba livia*) of unknown sex (450–550 g) were used. To test the performance of the stimulator, the stimulus-evoked potentials were detected in the hippocampus (Hp) area and the formatio reticularis medialis mesencephalic (FRM) nucleus was targeted for stimulation to induce the turning behaviour of pigeons, and both were selected referring to the previous work [24,25]. In this study, animal care and surgical procedures were approved by the Life Science Ethical Review Committee of Huanghuai University (no. LL20220001).

All surgeries were performed under general anaesthesia with 3% pelltobarbitalum natricum (0.12 ml/100 g body weight), and head feathers were shaved with 2% lidocaine injected subcutaneously as a further local anaesthetic. The animal was immobilized by a self-made brain stereotaxic device [26]. According to the atlas provided by Karten & Hodos [27], a stimulation electrode (diameter 100 µm stainless steel wire) was chronically implanted into the Hp (anterior–posterior (AP) 5.5 mm, medial–lateral (ML) 1.5 mm, dorsal–ventral (DV) 0–3 mm) for the detection experiment and into the FRM (AP 3.0 mm, ML 3.0 mm, DV 7.5–8.5 mm) for the regulation experiment. In addition, in the detection experiment, a detection electrode (16 channel, diameter 35 µm platinum-iridium wire, California Fine Wire Co., USA) was implanted simultaneously near the stimulation electrode.

Following surgery, all animals had at least 5–7 days to recover. In the detection experiment, the neural activities in the Hp area under electrical stimulation were recorded on free-moving pigeons with a Zeus™ digital signal processor (Bio-Signal Technologies, Nanjing City, China) under the default setting (30 kHz sampling rate). The recorded signals were filtered by a second-order Butterworth high-pass filter with a cut-off frequency of 0.5 Hz. In the regulation experiment, pigeon behaviour was modulated by stimulating the FRM to examine *in vivo* performance of the stimulator. Behavioural data were collected by animal behaviour analysis system (VisuTrack, Shanghai XinRuan Information Technology Co., Ltd, China). For the statistical analysis, statistical differences were evaluated by Wilcoxon signed-rank test, and the significance level was set to 5%, i.e. *p* < 0.05 was considered significant [28].

## 3. Results

### 3.1. General overview of the stimulator

The size of the stimulator (length × width × height) is 1.6 × 1.8 × 1.6 cm with an error below 0.1 cm (*n* = 20, figure 3*d*) and the weight of the stimulator is 4 ± 1 g (*n* = 20), where most of the error is caused by the encapsulation glue. The size and weight were suitable for most animals and far less than 3% of pigeon weight (figure 3*e*). The stimulator is capable of up to 1000 µA current (each phase) with 10 µA steps, the frequency limited from 5 to 200 Hz with the step of 5 Hz, and the PWR limited from 5% to 100% with the step of 5%. Although stimulation pulses with larger amplitude (maximum 2000 µA, each phase) and frequency (maximum 300 Hz) can be achieved under some conditions, the pulse waveform will exhibit large distortion.

The proposed stimulator is charged with a wired charger, which can be continuously charged and discharged 1000 times. The duration for charging is generally 20 ± 1 min (*n* = 20, HW-100400C01, Huawei Technologies Co., Ltd; output 5 V, 1 A). When the battery is fully charged, the proposed stimulator can be continuously used for about 10 h during rest (*n* = 20) and for about 6 h during different stimulation modes (*n* = 20, amplitude 500–900 µA, frequency 40–120 Hz, PWR 10–50%) (figure 3*f*). In addition, several experiments manipulating the distance between the handheld controller and the stimulator were performed to examine the working distance of the communication module (amplitude 600 µA, frequency 100 Hz, PWR 50%). The communication distance of stimulator can reach about 120 m in indoor environment and 150 m in outdoor environment (*n* = 20, 100% successful rate) (figure 3*g*). But no difference was shown in the results for free-moving animals whether in usual laboratory environments or an open environment.

### 3.2. *In vitro* evaluation of the stimulator

**Figure 4.**
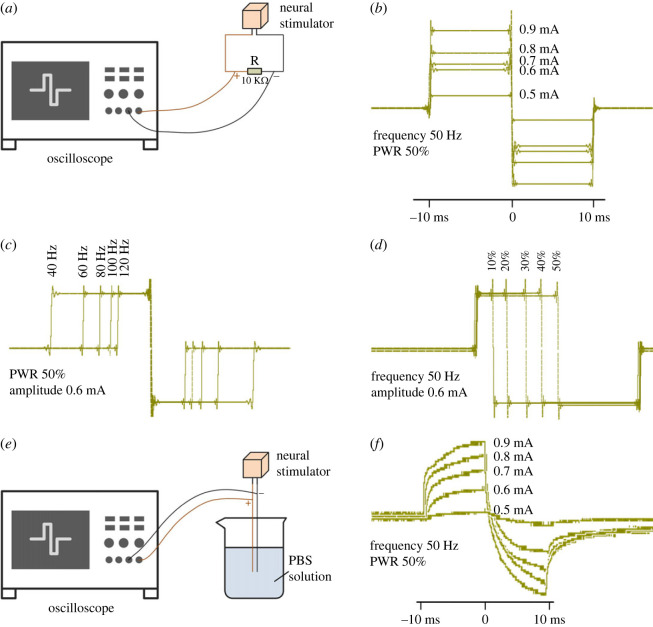
*In vitro* evaluation results. (*a*) Schematic of the stimulator connected to a resistance. (*b*) Pulse waveforms of different amplitude with both frequency and PWR set. (*c*) Pulse waveforms of a different frequency with both amplitude and PWR set. (*d*) Pulse waveforms of different PWR with both amplitude and frequency set. (*e*) Schematic of the stimulator connected to an electrode immersed in PBS solution. (*f*) Pulse waveforms of different amplitude with the stimulator tested by PBS solution, and both frequency and PWR set.

A 10 kΩ resistance is connected in series at the output channel of the stimulator (figure 4*a*) to evaluate the properties of the stimulator, where the impedance value is optional and other resistance is also acceptable. Stimulation amplitude, frequency and PWR are three core parameters for output pulse, making an important impact on the stimulation effect. Thus, with the other two parameters fixed, we measured the output pulse when one of the parameters gradually increased. Figure 4*b*–*d* corresponds to the results of amplitude, frequency and PWR, respectively, depicting that the output pulse will change with the increase of parameter values. The measurement results are basically consistent with the parameter settings. Despite some slight differences between output pulses and the settings, the results will not be affected.

In addition, the PBS solution was applied to mimic *in vivo*-like environment, and we further tested the *in vitro* performance of the stimulator (figure 4*e*). As an example, figure 4*f* shows the variation of stimulation amplitude with parameter (frequency 50 Hz and PWR 50%). Note that the output waveform is the voltage signal, which is different from that in figure 4*b*, which is the current signal. This result also verifies the performance of the stimulator from the side.

### 3.3. *In vivo* evaluation of the stimulator

**Figure 5.**
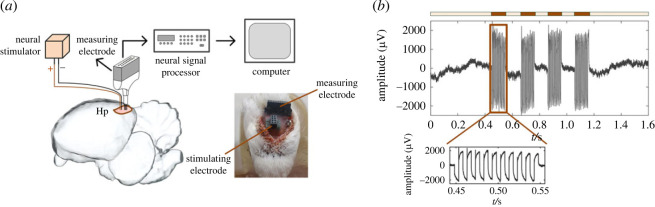
*In vivo* evaluation results with waveforms electrically evoked. (*a*) Schematic of the stimulator connected to an electrode implanted in the Hp area. The sub-figure is a photograph of the implanted micro-electrodes. (*b*) Raw data (0.5–5000 Hz) of the waveforms recorded from a pigeon, where the orange line indicates the electrical stimulation-evoked waveforms, with the stimulation parameters amplitude of 0.3 mA, frequency of 100 Hz and pulse-width ratio of 50%. The obviously increased waveform under the orange line indicates the efficiency of stimulation. The sub-figure is a partial enlarged view of the waveform. Note that the waveforms are a stimulation artefact, but not the neural response.

*In vivo* evaluation of the proposed stimulator is composed of the detection experiment testing and the regulation experiment testing. In the detection experiment, the stimulation electrode was connected to the stimulator depending on a wire, and the detection electrode was connected to the Zeus™ digital signal processor (figure 5*a*). When the biphasic pulses were delivered to the brain through the stimulation electrode, the waveforms were recorded by the detection electrode in the Hp of the pigeon. Zeus™ recorded a typical waveform (or artefact) under stimulation as shown in figure 5*b*, where the waveform amplitude was significantly increased by electrical stimulation, indicating a successful stimulation input and waveform output in the brain. Note that although the signal recorded here does not contain the neural responses evoked by stimulation, it clearly shows the artefact generated by stimulation, which fully illustrates the functionality of the stimulator.

**Figure 6.**
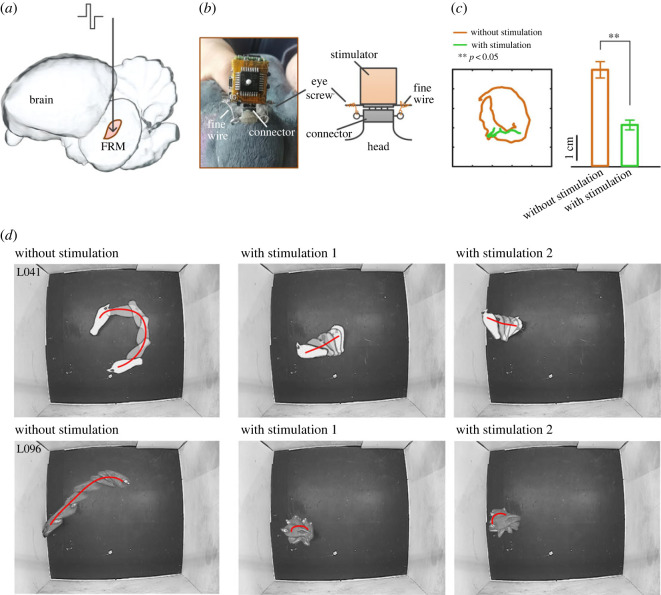
*In vivo* evaluation results using stimulation-controlled animal (animal robot). (*a*) Schematic of the FRM location when stimulated by stimulation pulse. (*b*) Both photograph and schematic of stimulator were fixed by eye screw. (*c*) Comparison of pigeon trajectories with and without stimulation. The trajectories without stimulation had significantly larger curvature radius than those with stimulation at the same time (*p* < 0.05, Wilcoxon signed-rank test). (*d*) Movement of two pigeons with or without stimulation. Note that the right figure shows movement of pigeons under the same stimulus twice.

To further estimate the performance of the stimulator, biphasic pulses were delivered through an electrode in the FRM of the pigeon (figure 6*a*). The electrode was connected with a connector (female header, Samtec Inc., New Albany, USA), which was fixed on the skull of the pigeon by dental cement. A corresponding connector (male header) on the stimulator is connected with the connector on the skull to transmit the stimulation pulses. To keep the stimulator from falling for a long time during the free-moving of subjects, a novel fixing method was designed (figure 6*b*), which includes four eye screws and two fine wires. Two eye screws are fixed on the skull, and the other two are fixed on the stimulator. Note that the eye screws on the skull are opposite to the eye screws on the stimulator (figure 6*b*). In the experiment, the connector of the stimulator is first inserted into the connector on the skull, and then fixed with fine wire. Our test results show that this fixation method can keep the stimulator on the pigeon head for at least 24 h (*n* = 5).

Figure 6*c* depicts an example of pigeon trajectories with or without stimulation in the same time duration (3 s), where the trajectories without stimulation were significantly higher than those with stimulation at the same time (*p* < 0.05, Wilcoxon signed-rank test). Figure 6*d* describes the behaviour of pigeons both nos. L041 and L096 exhibited with or without stimulation, where movements were random and irregular without stimulation, while reversed by the stimulus as the pigeon rapidly exhibited rotation or lateral displacement, which could be induced repeatedly with the same stimulus parameter. Although the final effect of stimulation may sometimes be affected by other factors, such as implantation site and surgery, the correct output of the stimulator pulse was also of great importance. The results further verify the practical function of the proposed stimulator.

## 4. Discussion

This present study proposed a wireless-controlled cubic neural stimulator for free-moving animals, with a size of 1.6 × 1.8 × 1.6 cm (error 0.1 cm), weight (including battery, etc.) of 4 ± 1 g and communication distance of approximately 150 m (open environment). Moreover, the charging duration of about 20 min can enable the stimulator to work continuously for 6 h during most stimulation modes. The flexible PCB was applied to print the circuit board which realizes a light weight, and a novel cube structure was designed to achieve a low volume. A commercial ZigBee module is used for wireless communication between the handheld controller and stimulator. The *in vitro* and *in vivo* testing results have depicted the functionality and practicability of the proposed stimator. More importantly, the stimulator is made of ordinary electronic components, which makes it acceptable to most laboratories at a low cost. Therefore, large communication distance, small volume, light weight and low cost make it suitable for most free-moving animals, especially for birds.

Electrical stimulation applied to stimulate specific brain regions of animals to evoke movement has a long history. As early as 2002, Talwar *et al*. [28] produced the first example with the neural stimulator where a rat was controlled to steer, jump or climb in several environmental scenarios. Despite the great progress achieved, its development has been hindered by a myriad of technological challenges, among which the stimulator is one of them. At the least, an electrode and a stimulator must be comprised to introduce artificial information into the nervous system. As the stimulator delivers electrical pulses on demand via control logic to the brain, all modules should be carried on the animals for the wireless stimulator, involving the pulse generating module, communication module, power supply module, etc. Therefore, it is critical to reach a balance in the volume, weight, working time and communication distance [29,30], which is also the core technical challenge for the wireless stimulator.

Table 1 summarizes the properties of the proposed stimulator, which is compared with state-of-the-art wireless neural stimulators. These stimulators could eliminate tethering wires to enable the unrestrained movement of the animals typically relying on wirelessly controlled and battery-powered stimulators mounted on the head. However, in comparison with other state-of-the-art devices, the flexible PCB technology and a cube structure were applied in the proposed stimulator, which provides several advantages. First, the flexible PCB has the advantages of high wiring density, light weight, thin thickness and good bending. Second, the cube structure can greatly release the volume of the stimulator without changing the area of the circuit board. Third, the battery is wrapped inside the circuit board, which makes the overall structure more stable. Although the capacity of the battery is limited, 100 mA h is sufficient for most experiments, which can support the continuous work of the stimulator about 6 h (amplitude 500–900 µA, frequency 40–120 Hz, PWR 10–50%).

**Table 1.** Comparison between the proposed and other wireless neural stimulators.

parameter	ref. [12]	ref. [15]	ref. [16]	ref. [18]	ref. [19]	this work
size (mm^3^)	48 × 23 × 19	20 × 40 × 5	29 × 26 × 8	26 × 16 × 9	36 × 22 × 7.5	16 × 18 × 16
weight (g)	28	9	5.9	9	40	4^a^
packaging	none	none	liquid crystal polymer	none	none	ethoxyline resin
number of channels	8	8	4	8	4	8
stimulation mode	voltage	current	current	voltage	voltage/current	current
communication	FM	ZigBee	ZigBee	FSK	Bluetooth	ZigBee
powering	Li battery (160 mA h)	Li battery (600 mA h)	Li battery (90 mA h)	Li battery (120 mA h)	Li battery (120 mA h × 2)	Li battery (50 mA h × 2)
power consumption	82.5 mW	165 mW	15.2 mW	51.8 mW	222 mW	46 mW

^a^Weight including battery.

Compared with the rigid PCB, the flexible PCB is easy to damage. So the stimulator needs to be encapsulated to extend its service life. Moreover, due to the easy bending characteristics of flexible PCB, the stimulator has the potential to be constructed into any shape, where the cube structure proposed in this study is only one of the feasible schemes. In the proposed stimulator, a ZigBee integrated circuit was applied, which limited the shape and size of our stimulator to some extent. However, in establishing the stimulator, if without rich production experience, a commercial integrated circuit is recommended first, which can not only reduce the production time but also improve the stability of the stimulator. For the fixation of the stimulator, the traditional stimulators are generally carried on the back by backpack, which may be more suitable for mammals. While for birds, the backpack can distract the subject's attention or cause emotional distress [31]. We found that it can be avoided if the stimulator is fixed on the head. To make the fixation more firm, a novel method was proposed. Through our observation, this method can not only fix the stimulator for a long time (greater than 24 h), but also will not affect the movement of pigeons, including flight.

For components, the proposed stimulator uses mature, economical, easy-to-get and easy-to-assemble parts. Although more recent components could help to achieve the low size and weight of the system further, this can be an advantage in certain conditions, such as low price, easy access and high maturity. More importantly, the novel cube structure design can make the stimulator enough space to accommodate these components. We also hope that the design can provide a reference for peers to help them design smaller, lighter and more powerful neural stimulators. In the future, we will pay more attention to further update the proposed stimulator so that it can be more perfectly presented to users.

## 5. Conclusion

In conclusion, a small (1.6 × 1.8 × 1.6 cm), light in weight (4 g) and multi-channel (eight-channel) wireless electrical stimulator for free-moving animals was presented in this study. Distinguished from traditional neural stimulators, flexible PCB technology was applied to establish a novel cube structure to obtain the low weight and low volume of the stimulator, where a structure with battery inside and circuit board outside was used, which makes it have high stability. A ZigBee integrated circuit is used for wireless communication between the handheld controller and stimulator, with the distance reaching about 150 m (open environment). The *in vitro* and *in vivo* testing results have verified its functionality, with the specific behaviour of pigeons induced repeatedly using the same stimulus parameter. Together, these results also help to promote research on the animal robots.

## Data Availability

The datasets are provided in the electronic supplementary material [32].

## References

[1] Barbruni GL, Ros PM, Demarchi D, Carrara S, Ghezzi D. 2020 Miniaturised wireless power transfer systems for neurostimulation: a review. IEEE T. Biomed. Circ. S. **14**, 1160-1178. (10.1109/TBCAS.2020.3038599) 33201828

[2] Shepherd RK, Villalobos J, Burns O, Nayagam DAX. 2018 The development of neural stimulators: a review of preclinical safety and efficacy studies. J. Neural Eng. **15**, 041004. (10.1088/1741-2552/aac43c) 29756600 PMC6049833

[3] Michelson RP. 1971 Electrical stimulation of the human cochlea: a preliminary report. Arch. Otolaryngol. **93**, 317-323. (10.1001/archotol.1971.00770060455016) 5100631

[4] Weiland JD, Humayun MS. 2008 Visual prosthesis. Proc. IEEE **96**, 1076-1084. (10.1109/JPROC.2008.922589)

[5] Benabid AL, Pollak C, Gervason C, Hoffmann D, Gao DM, Hommel H, Perret JE, de Rougemont J. 1991 Long-term suppression of tremor by chronic stimulation of the ventral intermediate thalamic nucleus. Lancet **337**, 403-406. (10.1016/0140-6736(91)91175-t) 1671433

[6] Kozielski KL, Jahanshahi A, Gilbert HB, Yu Y, Erin Ö, Francisco D, Alosaimi F, Temel Y, Sitti M. 2021 Nonresonant powering of injectable nanoelectrodes enables wireless deep brain stimulation in freely moving mice. Sci. Adv. **7**, eabc4189. (10.1126/sciadv.abc4189) 33523872 PMC7806222

[7] Sun C, Zheng NG, Zhang XL, Chen WD, Zheng XX. 2013 Automatic navigation for rat-robots with modeling of the human guidance. J. Bionic Eng. **10**, 46-53. (10.1016/S1672-6529(13)60198-5)

[8] Freeman DK et al. 2017 A sub-millimeter, inductively powered neural stimulator. Front. Neurosci. **11**, 659. (10.3389/fnins.2017.00659) 29230164 PMC5712043

[9] Piech DK et al. 2020 A wireless millimeter-scale implantable neural stimulator with ultrasonically powered bidirectional communication. Nat. Biomed. Eng. **4**, 207-222. (10.1038/s41551-020-0518-9) 32076132

[10] Ortiz-Catalan M. 2020 Ultrasound-powered tiny neural stimulators. Nat. Biomed. Eng. **4**, 144-145. (10.1038/s41551-020-0521-1) 32076131

[11] Fee MS, Leonardo A. 2001 Miniature motorized microdrive and commutator system for chronic neural recording in small animals. J. Neurosci. Meth. **112**, 83-94. (10.1016/s0165-0270(01)00426-5) 11716944

[12] Xu S, Talwar SK, Hawley ES, Li L, Chapin JK. 2004 A multi-channel telemetry system for brain microstimulation in freely roaming animals. J. Neurosci. Meth. **133**, 57-63. (10.1016/j.jneumeth.2003.09.012) 14757345

[13] Micco DJ. 1977 Lightweight, multi-contact, slip-ring commutator for recording and stimulation with small animals. Brain Res. Bull. **2**, 499-502. (10.1016/0361-9230(77)90060-0) 606345

[14] Hentall ID. 2013 A long-lasting wireless stimulator for small mammals. Front. Neuroeng. **6**, 8. (10.3389/fneng.2013.00008) 24130527 PMC3795361

[15] Alam M, Chen X, Fernandez E. 2013 A low-cost multichannel wireless neural stimulation system for freely roaming animals. J. Neural Eng. **10**, 066010. (10.1088/1741-2560/10/6/066010) 24162159

[16] Yun S et al. 2019 Remote-controlled fully implantable neural stimulator for freely moving small animal. Electronics **8**, 706. (10.3390/electronics8060706)

[17] Ding CQ. 1994 Application of radiotelemetry in bird research. Bull. Biol. **29**, 10-12.

[18] Yang J, Huai R, Wang H, Lv C, Su X. 2015 A robo-pigeon based on an innovative multi-mode telestimulation system. Bio-Med. Mater. Eng. **26**, S357-S363. (10.3233/BME-151323) 26406024

[19] Ye X, Wang P, Liu J, Zhang S, Jiang J, Wang Q, Chen W, Zheng X. 2008 A portable telemetry system for brain stimulation and neuronal activity recording in freely behaving small animals. J. Neurosci. Meth. **174**, 186-193. (10.1016/j.jneumeth.2008.07.002) 18674564

[20] Perry DWJ, Grayden DB, Shepherd RK, Fallon JB. 2012 A fully implantable rodent neural stimulator. J. Neural Eng. **9**, 014001. (10.1088/1741-2560/9/1/014001) 22248468 PMC3373993

[21] Zhou H, Xu Q, He J, Ren H, Zhou H, Zheng K. 2011 A fully implanted programmable stimulator based on wireless communication for epidural spinal cord stimulation in rats. J. Neurosci. Meth. **204**, 341-348. (10.1016/j.jneumeth.2011.10.028) 22085835

[22] Millard RE, Shepherd RK. 2007 A fully implantable stimulator for use in small laboratory animals. J. Neurosci. Meth. **166**, 168-177. (10.1016/j.jneumeth.2007.07.009) PMC2001238 17897719

[23] Shim S et al. 2019 A handheld neural stimulation controller for avian navigation guided by remote control. Bio-Med. Mater. Eng. **1**, 1-11. (10.3233/BME-191070) 31640081

[24] Zhao K, Wan H, Shang ZG, Liu XY, Liu L. 2019 Intracortical microstimulation parameters modulate flight behavior in pigeon. J. Integr. Neurosci. **18**, 23-32. (10.31083/j.jin.2019.01.14) 31091845

[25] Liu XY, Wan H, Chen XM, Shang ZG, Shi L, Li S, Chen X, Nie JJ. 2017 Response properties of place cells in the hippocampus of freely moving pigeons. Sci. Sin. Vitae **47**, 292-304. (10.1016/j.bbr.2008.03.023)

[26] Liu XY, Ping YN, Wang DY, Xie H, Shi L. 2022 Development of digital stereotaxic instrument for pigeons (*Columba livia*). J. Bionic Eng. **19**, 1003-1013. (10.1007/s42235-022-00194-0)

[27] Karten H, Hodos W. 1967 A stereotaxic atlas of the brain of the pigeon (Columba livia). Baltimore, ML: Johns Hopkins Press.

[28] Talwar SK, Xu S, Hawley ES, Weiss SA, Moxon KA, Chapin JK. 2002 Behavioural neuroscience: rat navigation guided by remote control. Nature **417**, 37-38. (10.1038/417037a) 11986657

[29] Bijak M, Schmoll M, Jarvis JC, Unger E, Lanmüller H. 2020 MiniVStimA: a miniaturized easy to use implantable electrical stimulator for small laboratory animals. PLoS ONE **15**, e0241638. (10.1371/journal.pone.0241638) 33125415 PMC7598460

[30] Samiei A, Hashemi H. 2020 Energy efficient neural stimulator with dynamic supply modulation. Electron. Lett. **57**, 173-174. (10.1049/ell2.12024)

[31] Zhou ZY, Liu DH, Sun H, Xu WB, Tian XM, Li XY, Cheng H, Wang ZL. 2021 Pigeon robot for navigation guided by remote control: system construction and functional verification. J. Bionic. Eng. **18**, 184-196. (10.1007/s42235-021-0013-3)

[32] Liu XY. 2022 Wireless-controlled cubic neural stimulator for free-moving animals. *Dryad Digital Repository*. (10.5061/dryad.cz8w9gj6w) PMC9974298 36866076

